# Retrospective Analysis and Cross-Validated Forecasting of West Nile Virus Transmission in Italy: Insights from Climate and Surveillance Data

**DOI:** 10.3390/tropicalmed10110305

**Published:** 2025-10-27

**Authors:** Francesco Branda, Mohamed Mustaf Ahmed, Dong Keon Yon, Giancarlo Ceccarelli, Massimo Ciccozzi, Fabio Scarpa

**Affiliations:** 1 Unit of Medical Statistics and Molecular Epidemiology, Università Campus Bio-Medico di Roma, 00128 Rome, Italy; m.ciccozzi@unicampus.it; 2 Faculty of Medicine and Health Sciences, SIMAD University, Mogadishu 2526, Somalia; momustafahmed@simad.edu.so; 3 Center for Digital Health, Medical Science Research Institute, Kyung Hee University College of Medicine, Seoul 05278, Republic of Korea; yonkkang@gmail.com; 4 Department of Precision Medicine, Kyung Hee University College of Medicine, Seoul 05278, Republic of Korea; 5 Department of Pediatrics, Kyung Hee University College of Medicine, Seoul 05278, Republic of Korea; 6 Department of Public Health and Infectious Diseases, University of Rome Sapienza, 00185 Rome, Italy; giancarlo.ceccarelli@uniroma1.it; 7 Department of Biomedical Sciences, University of Sassari, 07100 Sassari, Italy; fscarpa@uniss.it

**Keywords:** West Nile Virus, predictive modelling, XGBoost, Italy, machine learning, outbreak detection, climate, epidemiology

## Abstract

**Background.** West Nile Virus (WNV) represents a significant public health concern in Europe, with Italy—particularly its northern regions—experiencing recurrent outbreaks. Climate variables and vector dynamics are known to significantly influence transmission patterns, highlighting the need for reliable predictive models to enable timely outbreak detection and response. **Methods.** We integrated epidemiological data on human WNV infections in Italy (2012–2024) with high-resolution climate variables (temperature, humidity, and precipitation). Using advanced feature engineering and a gradient boosting framework (XGBoost), we developed a predictive model optimized through time-series cross-validation. **Results.** The model achieved high predictive accuracy at the national level (R^2^ = 0.994, MAPE = 5.16%) and maintained robust performance across the five most affected provinces, with R^2^ values ranging from 0.896 to 0.996. SHAP analysis identified minimum temperature as the most influential climate predictor, while maximum temperature and rainfall demonstrated considerably weaker associations with case incidence. **Conclusions.** This machine learning approach provides a reliable framework for forecasting WNV outbreaks and supports evidence-based public health responses. The integration of climate and epidemiological data enhances surveillance capabilities and enables informed decision-making at regional and local levels.

## 1. Introduction

West Nile Virus (WNV) represents a significant and evolving public health challenge globally, with a growing impact also in Europe and, in particular, in the Mediterranean basin area [1]. This zoonotic virus, belonging to the Flaviviridae family, is mainly transmitted to humans through the bite of the key vector mosquitoes of the genus *Culex*, which acquire the virus by biting birds, the main natural reservoirs in the epidemiological chain [2]. As summarized in Table 1, although most human infections are asymptomatic or present mild symptoms, a significant proportion of individuals may develop West Nile Fever (WNF), a usually self-limiting febrile illness, while a minority evolve to severe neuroinvasive forms (WNND) involving encephalitis, meningitis, or acute flaccid paralysis, with high mortality and long-term morbidity rates [3,4].

In Europe, the spread of WNV has been reported in an increasing number of countries over the past two decades. A recent systematic review reported 5765 confirmed human cases across 19 Mediterranean countries between 2010 and 2023, of which 4546 occurred in Southern Europe. Italy (2280 cases) and Greece (1868 cases) were identified as the most affected countries, highlighting the persistence of local foci and recurrent transmission cycles [8]. Within this broader regional context, Italy—particularly the northern regions—represents one of the most affected areas, where the virus has become endemic and causes recurrent annual epidemics. The first official report of WNV in the country dates to 1998, with documented cases in Tuscany [9]. After a period of inactivity, the virus re-emerged significantly in 2008, affecting the Po Delta and several provinces in neighbouring regions, with evidence of co-circulation of different genetic lineages (Lineage 1 and 2), confirming a complex and dynamic epidemiological picture [10,11]. Recent analyses further highlighted the role of Lineage 2 in sustaining outbreaks in Italy, showing how viral genetics interact with environmental drivers to influence incidence trends [12].

The persistence and annual recurrence of WNV testify that it is no longer a sporadic event but an established and expanding threat that requires ongoing surveillance interventions, constant monitoring, and integrated control strategies [1]. Recent surveillance data confirm the value of a One Health approach: viral RNA has been repeatedly detected in *Culex* mosquitoes and wild birds, while seroprevalence rates up to 30% have been documented in horses and avian sentinels in endemic areas, underscoring the importance of integrated human–animal–vector monitoring [8]. The Veneto region exemplifies the value of integrated surveillance in anticipating viral circulation and clarifying the role of avian hosts in the enzootic cycle within Italy’s endemic areas, where during the unprecedented 2022 epidemic more than 530 human infections were reported, alongside over 90,000 mosquitoes and 2000 wild birds tested, which revealed infection rates close to 5% and 8%, respectively. This explosive circulation was fueled by the simultaneous presence of WNV Lineages 1 and 2 and by co-circulation with Usutu virus (USUV), a combination rarely documented elsewhere in Europe [13].

Climate change, particularly rising average temperatures and changing rainfall patterns, facilitates the geographic and temporal expansion of *Culex* vectors, prolonging the transmission season and increasing the risk of outbreaks [14,15]. Urbanization and modification of natural ecosystems, resulting in altered bird and mosquito habitats, also play a crucial role in increasing the vulnerability of human populations [16].

Predictive modelling plays a central role in supporting public health, enabling the anticipation of risk periods and efficient targeting of resources. Traditional epidemiological models rely on the integration of meteorological data, including temperature, humidity, precipitation, and entomological and epidemiological data to estimate transmission dynamics [17]. Such models have been shown to improve predictive ability compared to clinical surveillance data alone, especially by incorporating time delays between favourable environmental conditions and human case emergence/occurrence of human cases [18]. However, the complexity of interactions between vectors, hosts, and the environment, as well as the influence of anthropogenic factors, necessitates the development of more sophisticated models capable of capturing nonlinearities, spatial and temporal variations, and the inherent uncertainty in the data [19]. The use of machine learning and artificial intelligence-based approaches is opening up new perspectives for WNV modelling, thanks to the ability to handle large masses of heterogeneous data and detect complex patterns [20]. These methods can integrate climate, entomological, demographic, and surveillance data to produce more accurate and timely predictions, which are critical for the activation of targeted and timely interventions. Despite this, data availability and quality remain a major limitation, as does the need for rigorous validation and interpretability of models.

In this context, the present study aims to develop an advanced predictive model for WNV in Italy, by integrating detailed climatic and epidemiological data. Specifically, Section 2 describes the data sources and the XGBoost modelling approach. Section 3 presents the results, showing temporal trends, age-related patterns, species distribution, and strong model performance across provinces. Section 4 discusses implications for public health, highlighting how climate and vector dynamics influence outbreak risk.

## 2. Materials and Methods

Epidemiological data on WNV cases were collected from PDF bulletins compiled by Italian national health authorities (https://www.epicentro.iss.it/westNile/bollettino, accessed on 10 September 2025). The dataset [21] is structured to summarize the results of integrated surveillance in Italy, including data on virus circulation in humans (neuroinvasive cases, febrile cases, and cases in blood donors), in animals (horses, target birds, and wild birds), and in vectors (mosquitoes), as outlined in the National Prevention, Surveillance and Response Plan for Arboviral Diseases 2020–2025 available at the link: https://www.statoregioni.it/media/2371/p-1-csr-rep-n-1-15gen2020.pdf (accessed on 10 September 2025). In this context, the term target birds refers to receptive resident avian species routinely sampled for the early detection of West Nile Virus circulation, as defined in Section 3.5.1 of the national surveillance plan for arboviruses in Italy. These species include the Eurasian magpie (*Pica pica*), hooded crow (*Corvus corone cornix*), and Eurasian jay (*Garrulus glandarius*). They are selected based on their ecological role as competent hosts, their abundance, and their territorial behaviour, which make them suitable indicators of viral activity in the environment prior to human or equine cases. Note that data on human cases are available from 2012, whereas data for animals and vectors are available from 2017. Moreover, no personally identifiable information was used, and all health data were aggregated at the administrative level in accordance with applicable privacy regulations.

The analysis was performed across multiple spatial scales. While descriptive summaries of WNV circulation were generated at the national and regional levels, predictive modelling and validation were conducted at the provincial level to accurately capture local transmission dynamics. The model operates on a daily temporal resolution, but epidemiological data are reported in weekly aggregates. To reconcile these scales and enable the derivation of short-term temporal features, weekly case counts were disaggregated uniformly across the corresponding days, preserving the total incidence while providing a high-resolution daily time series. This disaggregation was essential for computing features such as daily case differences, rolling means, and rolling standard deviations, which underpin the model’s predictive capability. Observations from equines, birds, and mosquitoes were included solely for descriptive purposes within the One Health surveillance framework and were not incorporated as predictor variables for human cases.

Meteorological data, including daily maximum and minimum temperature, humidity and precipitation, were obtained from the Open-Meteo Archive API (https://open-meteo.com, accessed on 10 September 2025) by querying a grid of geographic coordinates spanning the Italian territory. A custom R script automated data retrieval for each lat-lon pair within this bounding box for the period 2017–2024. These daily variables were selected because they are widely used in epidemiological studies [22,23] on WNV and are known to strongly influence mosquito dynamics and virus transmission. Although hourly data and additional variables such as atmospheric pressure, wind speed, and wind direction could provide more detailed information, they were not consistently available across all regions and years.

All preprocessing and initial data integration were performed using the R programming language (version 4.5.1) within RStudio (version 2025.05.1). The data pipeline included parsing and cleaning of case data with *dplyr*, date formatting with *lubridate*, and spatial aggregation of meteorological variables through grouping by date and geographic coordinates. Daily averages of temperature and precipitation were calculated, and weather data were merged with WNV case records by matching observations that shared the same calendar date. This procedure ensured temporal alignment between meteorological variables and incidence data, retaining only records present in both datasets.

After data integration, advanced feature engineering and predictive modelling were conducted in Python (version 3.11) using the *pandas*, *numpy*, *scikit-learn*, *matplotlib*, and *xgboost* libraries. A comprehensive overview of all explanatory variables used in model training—including their source, spatial and temporal resolution, and calculation—is provided in Table A1. Meteorological features were augmented with derived variables, including 7-day rolling averages and cumulative precipitation, diurnal temperature range, and cyclical encodings of the day of the year using sine and cosine transformations to capture seasonal dynamics in a continuous manner (i.e., treating day 1 and day 365 as adjacent). Additional predictors included temporal lags and moving statistics (mean, standard deviation) of new case counts and average temperature at 7-, 14-, 21-, and 28-day intervals. Short-term trends were captured through daily and weekly differences in incidence. The target variable was defined as the natural logarithm of (1 + new daily WNV cases) to stabilize variance and accommodate zero values. An 80/20 time split was applied, meaning that 80% of the time series was used for training and the most recent 20% was reserved for testing, so that the model evaluation was always performed on future, unseen data. Additional tests with alternative splits (70/30 and 90/10) produced comparable performance, confirming the robustness of the results. Specifically, a grid search over combinations of number of estimators, learning rate, and maximum tree depth was performed using a five-fold TimeSeriesSplit, a cross-validation method specifically designed for time-ordered data. Unlike standard k-fold cross-validation, which randomly splits the data, TimeSeriesSplit respects the chronological order: for each fold, the model is trained on all available past observations and evaluated on subsequent future data. This approach prevents data leakage from the future into the training set and provides a more realistic assessment of the model’s predictive performance in prospective scenarios, which is particularly important for epidemiological time series [24].

Model performance was evaluated using multiple complementary metrics to provide a comprehensive assessment of predictive accuracy and reliability. Metrics such as the Mean Absolute Error (MAE), Root Mean Squared Error (RMSE), Median Absolute Error (MedAE), and Mean Absolute Percentage Error (MAPE) measure the accuracy of prediction errors, that is, how close the model estimates are to the observed values. The Coefficient of Determination (R^2^) quantifies how much of the variance in the observed data is explained by the model, while the Pearson correlation coefficient (r) indicates how well predictions follow the linear trend of the observed data. Formal definitions of these standard metrics are provided in Appendix A. Moreover, feature relevance was quantified using both internal XGBoost metrics (gain, cover, frequency) and permutation importance. To understand the effect of key climatic variables on predictions, Partial Dependence Plots (PDPs) and SHAP values were computed. PDPs describe the marginal effect of each variable on predicted outcomes, while SHAP values provide both global and local contributions of features, capturing non-linear and interaction effects.

We also used the Continuous Ranked Probability Score (CRPS) to evaluate the quality of the probabilistic forecasts. The CRPS compares the entire predicted probability distribution to the observed outcome, integrating information on both calibration, i.e., how well predicted probabilities match observed frequencies, and sharpness, i.e., how concentrated the predictive distribution is. It can be interpreted as a probabilistic analogue of the MAE, quantifying the average distance between the predicted cumulative distribution function (CDF) and the empirical CDF of the observation. Assuming a Gaussian predictive distribution $N(\mu_{i},\sigma_{i})$ for each observation *i*, the CRPS is computed as

(1) $$\mathrm{CRPS}\left(N\left(\mu_{i},\sigma_{i}\right),y_{i}\right)=\sigma_{i}\left[\omega_{i}\left(2\Phi (\omega_{i})-1\right)+2\phi \left(\omega_{i}\right)-\frac{1}{\sqrt{\pi }}\right],$$

where $\mu_{i}$ and $\sigma_{i}$ are the predicted mean and standard deviation for observation *i*, $\omega_{i}=\frac{y_{i}-\mu_{i}}{\sigma_{i}}$, and Φ(·) and ϕ(·) denote the cumulative and probability density functions of the standard normal distribution, respectively. Lower CRPS values indicate higher accuracy and better calibration of probabilistic predictions. This metric is widely adopted in meteorological and epidemiological forecasting contexts, as it jointly rewards precision and well-calibrated uncertainty estimates [25].

## 3. Results

### 3.1. Summary of the Situation

**Figure 1:**
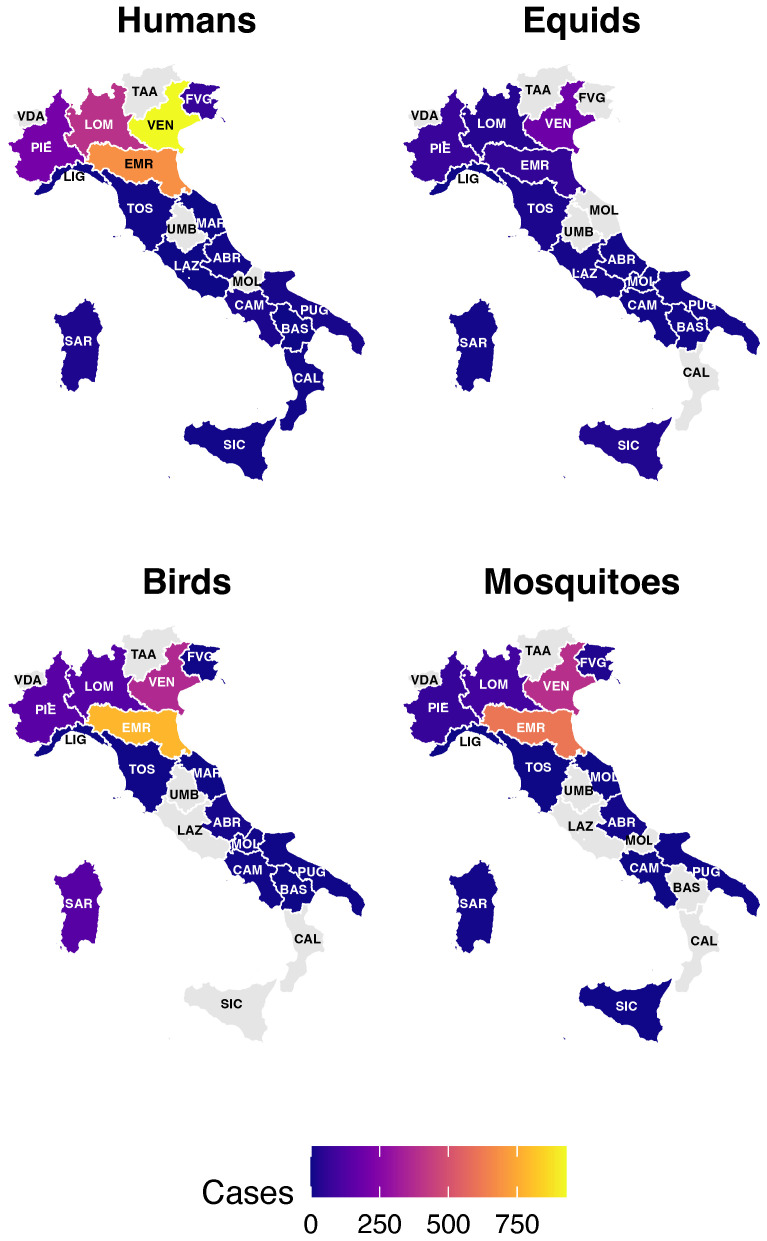
Geographic distribution of West Nile Virus cases in Italy between 2012 and 2024, grouped by supervised category: human population, equids, avifauna (wild and target), and mosquitoes. Abbreviations: LOM (Lombardy), VEN (Veneto), EMR (Emilia-Romagna), PIE (Piedmont), TOS (Tuscany), LAZ (Lazio), PUG (Apulia), SIC (Sicily), SAR (Sardinia), FVG (Friuli Venezia Giulia), MAR (Marche), ABR (Abruzzo), CAL (Calabria), BAS (Basilicata), CAM (Campania), MOL (Molise), UMB (Umbria), LIG (Liguria), TRV (Trentino-Alto Adige/Südtirol), VDA (Aosta Valley).

Aggregate analysis at the regional level shows that WNV has a highly heterogeneous geographic distribution, with some regions in northern Italy acting as true multi-host viral circulation hotspots, as shown in Figure 1. Veneto emerges as the region with the highest total number of reported cases in all host categories. Between 2012 and 2024, 928 human cases were reported; during 2017–2024, 194 cases were reported in horses, 368 in birds, and 390 in mosquito pools. It is followed by Emilia-Romagna, also heavily affected, with 685 human (2012–2024), 60 equine, 782 avian, and 613 entomological cases between 2017 and 2024, suggesting sustained and well-established transmission in the territory. Lombardy and Piedmont also show consistent numbers, particularly in terms of human cases (397 and 215, respectively, between 2012 and 2024) and animal surveillance between 2017–2024. The Central and Southern regions show more fragmented viral activity, with sporadic human outbreaks and a more discontinuous presence in the vector and animal components. However, the presence of the virus should also be reported in island regions such as Sardinia (23 human, 8 equine, 141 avian, 8 entomological cases) and Sicily, albeit with lower numbers. The number of infected birds in Emilia-Romagna (782 cases between 2017 and 2024) is an important early indicator, suggesting that the virus is actively circulating in the environment prior to the detection of human cases.

### 3.2. Descriptive Characteristics of the Infections

**Figure 2:**
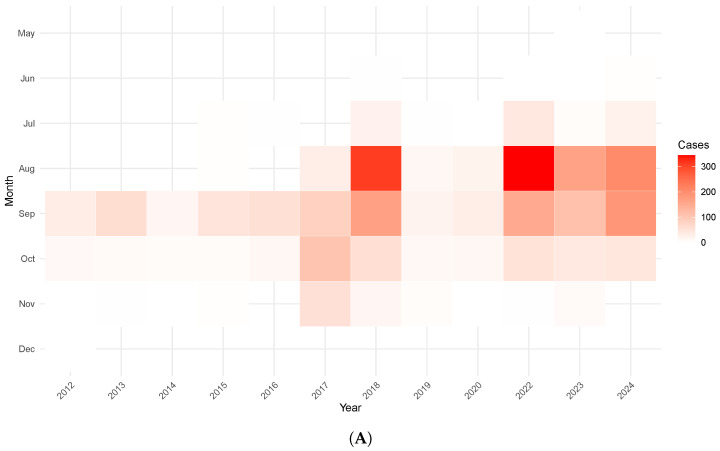

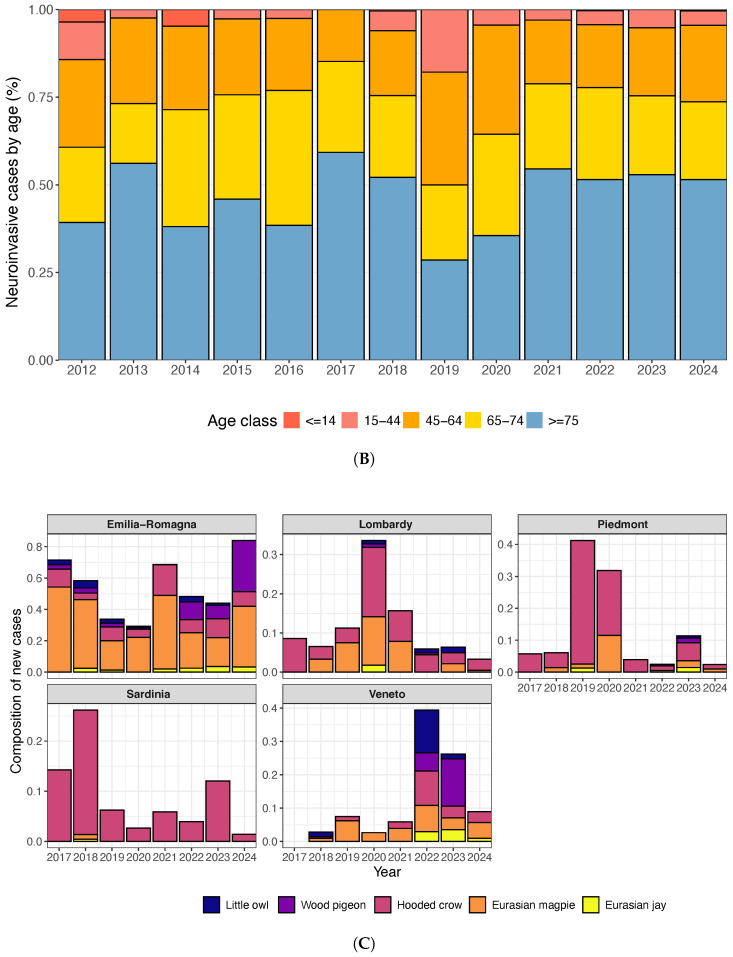
(**A**) Monthly and annual distribution of human cases of WNV infection in Italy. (**B**) Age distribution of human neuroinvasive WNV infections. (**C**) Top 5 target bird species affected and their distribution in the 5 most affected regions.

The temporal distribution of human cases of WNV infection in Italy shows a marked seasonality concentrated between August and September (Figure 2A). In particular, August records the highest values, with a maximum of 345 cases in 2022 and 311 in 2018, while September follows with a significant number of cases, reaching up to 186 in 2024. In the following months, there is a decrease in cases, while November and December show almost no cases, confirming the highly seasonal nature of the epidemic. Moreover, recent years, particularly since 2022, there has been a noticeable increase in the number of cases, in line with the findings of Loconsole et al. [26], who reported an abrupt rise in locally acquired WNV infections in Italy, including severe neuroinvasive forms. Regarding the distribution by age group (Figure 2B), the most affected population appears to be the elderly, with more than half of the neuroinvasive cases concentrated in people aged 75 or older (e.g., 52% in 2018 and 2023). Intermediate age groups between 45 and 74 also contribute significantly, while younger individuals, particularly those under 14, show a very low percentage of cases. These age-related patterns specifically describe the distribution of neuroinvasive WNV infections, highlighting the greater clinical vulnerability of older age groups to severe disease. However, this distribution reflects differences in clinical severity and healthcare-seeking behavior rather than necessarily indicating higher infection rates or increased transmission intensity in these age groups, as asymptomatic and mild infections are less likely to be diagnosed and reported.

An analysis of the WNV infections in target bird species from 2017 to 2024 (Figure 2C) reveals that the most affected species consistently include the Eurasian magpie (*Pica pica*), the hooded crow (*Corvus corone cornix*), the common wood pigeon (*Columba palumbus*), the little owl (*Athene noctua*), and the Eurasian jay (*Garrulus glandarius*). Emilia-Romagna stands out as the most impacted region, frequently reporting the highest case numbers across these species. For instance, cases of Eurasian magpie in Emilia-Romagna represent a substantial proportion of total bird infections yearly, reaching up to 54% in 2017 and maintaining a dominant share in subsequent years. Other regions such as Lombardy, Piedmont, Veneto, and Sardinia also contribute variably to infection counts, particularly for hooded crow and common wood pigeon.

### 3.3. Predictive Model Performance

**Figure 3:**
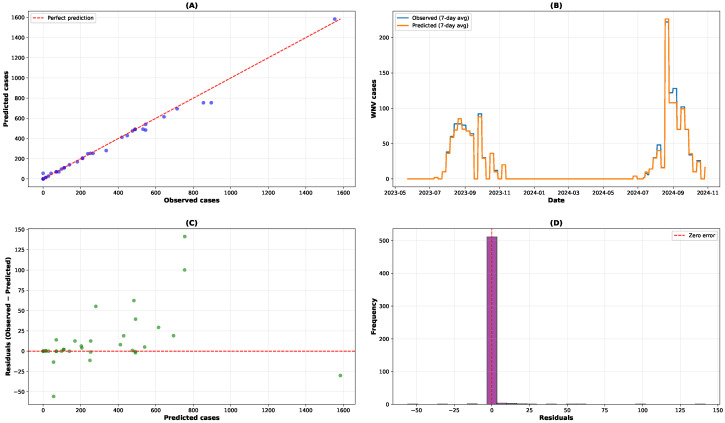
Comprehensive model performance. (**A**) Observed vs. predicted cases scatter plot, (**B**) temporal trends with 7-day rolling averages, (**C**) residual plot, and (**D**) distribution of prediction errors.

The predictive model demonstrated strong performance across all evaluation metrics, exhibiting reliable forecasting capabilities for WNV transmission dynamics in Italy. As shown in Figure 3, the model achieved accurate predictions of daily case counts, with good alignment between observed and predicted values throughout the test period.

The temporal trends (Figure 3A,B) show effective capture of seasonal patterns and outbreak dynamics, with the 7-day rolling averages of observed and predicted cases showing good correspondence across the evaluation period. The visualization focuses on the 2023–2024 period, which represents the test set following the temporal train–test split: the model was evaluated on the most recent 20% of the data (2023–2024) after being trained on the preceding 80% (2012–2022). This temporal validation strategy provides a realistic assessment of the model’s predictive performance on unseen future data, a key requirement for operational forecasting applications. The selected period includes substantial transmission activity, allowing for meaningful evaluation of the model’s ability to capture both seasonal patterns and outbreak dynamics.

**Figure 4:**
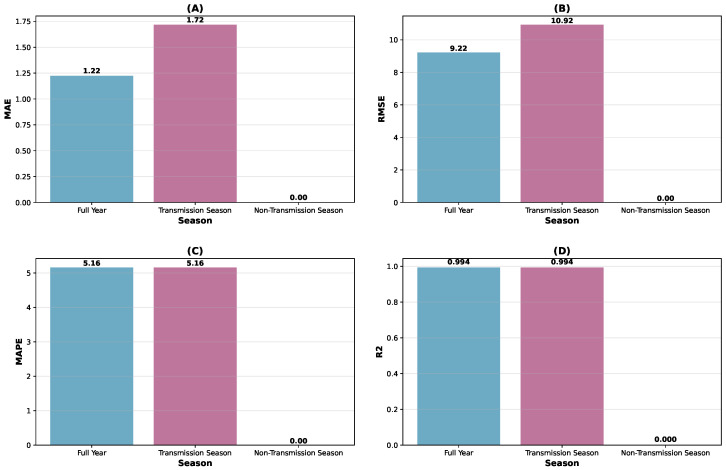
Seasonal performance comparison at national level. (**A**) MAE, (**B**) RMSE, (**C**) MAPE, and (**D**) R^2^ across full year, transmission season, and non-transmission season.

At the national level, the model achieved an R^2^ value of 0.994, indicating that 99.4% of the variance in observed WNV cases was explained by the model. As summarized in Figure 4, the MAE of 1.22 (Figure 4A) and RMSE of 9.22 (Figure 4B) reflect moderate prediction errors relative to the scale of case counts. The MAPE of 5.16% (Figure 4C) indicates that predictions deviated from observed values by approximately 5% on average, representing satisfactory accuracy for epidemiological forecasting applications. The R^2^ of 0.994 (Figure 4D) highlights the model’s strong explanatory power.

**Figure 5:**
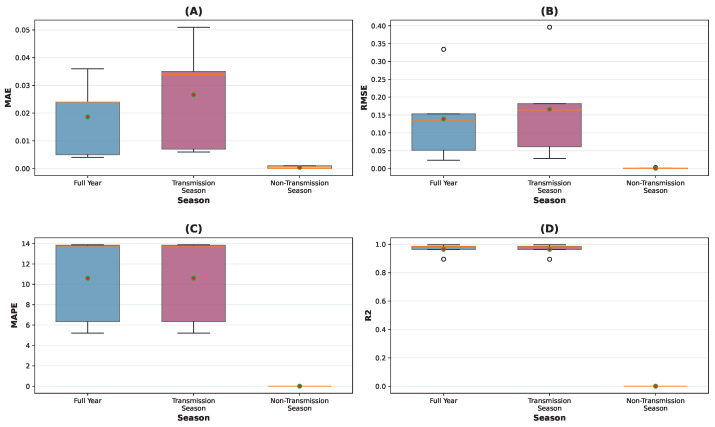
Provincial distribution of seasonal metrics. (**A**) MAE, (**B**) RMSE, (**C**) MAPE, and (**D**) R^2^ across different seasonal periods. The red and white dots represent individual data points, while the orange lines connect the median values for each seasonal period to illustrate trends across seasons.

Seasonal performance analysis (Figure 5) revealed consistent model performance during the transmission season (May–November), with R^2^ maintained at 0.994 and MAPE at 5.16%, despite higher case counts (mean observed: 29.33 vs. 20.92 annually). This suggests the model maintains its predictive capability during high-transmission periods when forecasting accuracy is particularly relevant for public health planning.

The provincial-level analysis further supported the model’s predictive capability at finer spatial scales. As summarized in Table 2, the model maintained good performance across all five most affected provinces, with R^2^ values ranging from 0.896 (Modena) to 0.996 (Verona). The MAE and RMSE values indicate reasonable estimation of daily case counts across provinces with varying epidemic intensities. The low Median AE values (0.000–0.001) across provinces suggest consistent performance, while Pearson correlation coefficients near 1.0 indicate strong linear agreement between predicted and observed values.

The seasonal performance at provincial level (Table 3) provides additional characterization of the model’s operational properties. All provinces maintained acceptable predictive accuracy during transmission seasons, with MAPE values ranging from 5.21% to 13.87%. The near-zero metrics during non-transmission seasons are consistent with appropriate handling of baseline periods with minimal case activity. The provincial distribution of seasonal metrics (Figure 5) further indicates consistent performance across different geographical contexts, with stable R^2^ values across provinces (Figure 5D).

The CRPS scores across provinces (Table 2) suggest reasonable uncertainty quantification, supporting the model’s potential utility for risk assessment applications. The model’s maintenance of predictive accuracy during both transmission periods and seasonal baselines, combined with its consistent performance across provincial contexts as shown in Figure 5, indicates it could serve as a useful component in WNV surveillance systems for targeted public health response.

### 3.4. Key Predictors of WNV Incidence

To comprehensively address feature importance in our XGBoost model, we conducted a SHAP analysis, which provides a unified measure of feature importance while accounting for complex interactions within the model. The SHAP analysis revealed a clear hierarchy of predictive features (Table 4). Short-term epidemiological trends were mainly determined by the daily variation in case (*new_cases_diff_1*) emerging as the most influential feature (SHAP importance = 0.288694). This was followed by the 7-day rolling mean of cases (*new_cases_rolling_mean_7*, SHAP importance = 0.042545), indicating that recent transmission dynamics are the primary drivers of model predictions.

Among climate variables, minimum temperature (*tmin*) was the most important climatic predictor (SHAP importance = 0.003564), ranking third overall among all features. Rainfall (*rain*) showed moderate influence (SHAP importance = 0.002972), while maximum temperature (*tmax*) demonstrated substantially lower importance (SHAP importance = 0.000096). The relative ranking of climate features based on SHAP importance was *tmin* (rank 3) > *rain* (rank 5) > *rain7d* (rank 14) > *tmax* (rank 16) > *temp_avg* (rank 22).

**Figure 6:**
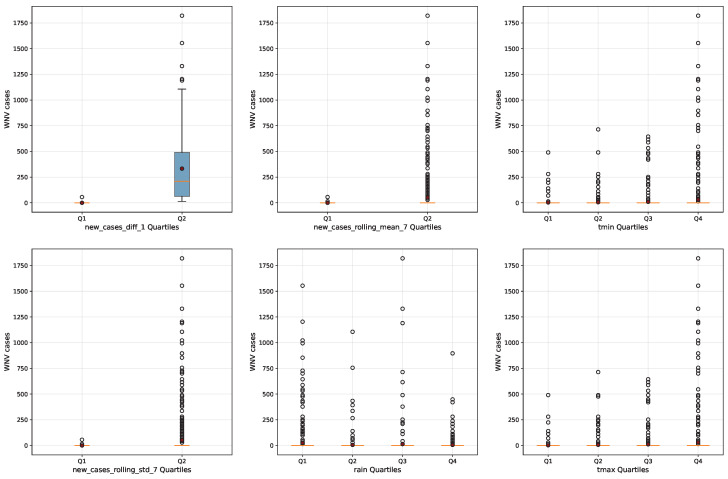
Distribution of WNV cases by top SHAP feature quartiles. The red and white dots represent individual data points, while the orange lines connect the median values for each seasonal period to illustrate trends across seasons.

Feature quartile analysis (Figure 6) has confirmed the SHAP and XGBoost results by illustrating how environmental and epidemiological drivers modulate WNV transmission. Mean case counts across minimum temperature quartiles increase sharply from the coolest (Q1: 3.26 cases) to the warmest quartile (Q4: 31.45 cases), supporting the threshold effect where temperatures above 20 °C drive higher transmission risk. Rainfall exhibits a U-shaped effect, with the highest mean cases observed under dry conditions (Q1: 25.46 cases) and reduced transmission during moderate rainfall (Q2: 5.60 cases), while very wet conditions also moderate cases (Q4: 5.48 cases). Maximum temperature quartiles show a 14.9-fold increase in mean cases from Q1 to Q4, but median values remain zero across all quartiles, highlighting the challenge of predicting rare outbreak events from continuous environmental data.

XGBoost internal metrics further quantify these patterns. The feature *new_cases_diff_1* accounts for the largest gain (28.25%) and extensive tree coverage (1935.6), with high frequency in model splits (320 trees). The 7-day rolling mean contributes a gain of 2.37 and cover of 49.3, while *tmin* shows smaller gain (0.135) but substantial cover (1881.5), indicating frequent use in trees. Permutation importance confirms this hierarchy: *new_cases_diff_1* exhibits the strongest effect (2.55 ± 0.16), followed by the 7-day rolling mean (0.249 ± 0.031), with *tmin* (−0.009 ± 0.031) and *rain* (−0.018 ± 0.015) showing smaller yet consistent contributions.

## 4. Discussion

The results of this study demonstrate that the integration of climatic and epidemiological data through advanced machine learning techniques can significantly improve the predictive capability for WNV outbreaks in Italy. The high accuracy and low error rates observed—particularly in the most affected provinces of northern Italy—support the feasibility of incorporating such models into national and regional early warning systems.

Temperature emerged as the most relevant climatic variable positively correlated with new cases, aligning with existing evidence that highlights the role of warmer temperatures in accelerating mosquito development and viral replication within vectors. This interpretation is consistent with previous observations in Italy, where interannual fluctuations in case counts (e.g., the apparent reduction in 2019–2020) were not indicative of viral disappearance, but rather reflected climatic variability, with spring and winter temperatures acting as key drivers of subsequent seasonal intensity [7].

The lack of significant association with humidity and only marginal correlation with precipitation may be due to the complex, nonlinear relationships these factors have with vector ecology, or to the temporal lag between rainfall and mosquito population surges. In our approach, we included temporal lags of meteorological variables (up to 28 days) to approximate delays in mosquito eclosion and life-cycle dynamics, which are important determinants of WNV transmission and can mediate the effects of humidity and precipitation. However, we did not explicitly model entomological processes. Future work incorporating a more biologically informed lag structure, parameterized on vector ecology, could improve the interpretability of precipitation and humidity effects and strengthen the ecological realism of predictive models.

Our findings are consistent with prior research suggesting that traditional surveillance alone may not be sufficient to anticipate outbreak emergence in a timely manner. Notably, the work of Farooq et al. [18] and Angelou et al. [20] has shown that machine learning models incorporating eco-climatic features significantly outperform conventional models in terms of outbreak forecasting. This study contributes to that growing body of evidence by providing a validated model trained on over a decade of national data, further supporting the application of artificial intelligence in real-time epidemiological applications. Moreover, the robust model performance at the provincial level is particularly encouraging. In areas such as Veneto and Emilia-Romagna, where viral circulation in both human and animal hosts is consistently high, the model achieved R^2^ values approximating 0.99. This granularity is essential for actionable public health interventions, especially considering the focal nature of WNV transmission. Recent European-scale projections provide additional support for integrating predictive modelling into long-term public health planning. Farooq et al. [27] used an ensemble climate modelling approach combined with XGBoost and socio-economic scenarios to estimate that the proportion of European land areas at risk for WNV could increase from 15% to 23–30% by 2040–2060, with up to a five-fold increase in outbreak risk under high-emission scenarios. Western Europe is expected to experience the most pronounced expansion, but currently low-risk regions may also be affected later in the century. Notably, their work identified spring climatic conditions—particularly maximum spring temperatures exceeding 15 °C and lower relative humidity—as strong early-warning signals for subsequent transmission intensity. Incorporating such climate-based projections and threshold signals into national models could enhance their strategic value, enabling anticipation of epidemiological shifts decades in advance. This aligns with our emphasis on operational integration of machine learning frameworks into surveillance systems and suggests that extending models to include downscaled climate projections may allow for robust, scenario-based forecasting.

In addition, beyond improving the prediction of human cases, our modelling approach has the potential to complement existing sylvatic cycle surveillance. The sylvatic cycle of WNV in Italy involves complex interactions between avian reservoirs and mosquito vectors, which sustain viral circulation independently of human infections. Current entomological and ornithological surveillance provides critical early signals of viral activity, but its spatial coverage is inherently limited and often subject to delays due to logistical constraints. By integrating climatic and epidemiological predictors, our model can identify periods and areas of heightened transmission risk, potentially indicating underlying sylvatic amplification even before human cases are detected. In this sense, predictive models could act as a complementary tool to traditional wildlife and vector monitoring, supporting targeted entomological sampling in high-risk areas and improving the overall sensitivity of early warning systems. This integration would be particularly valuable in regions where continuous vector and avian surveillance is not feasible, strengthening a One Health approach to arboviral surveillance.

Finally, while hourly weather data and additional variables such as dew point, atmospheric pressure, cloud coverage, UV index, wind speed and direction, reference evapotranspiration, or vapor pressure deficit could provide more detailed information, these data are not consistently available across all regions and years of our study period. The use of daily summaries ensured data completeness, consistency, and comparability. Nonetheless, incorporating higher-resolution weather data and a broader set of meteorological variables could further refine predictive models and should be the subject of future research aimed at improving WNV forecasting accuracy.

In line with our previous work through the ArboItaly project [28], which advocated for the integration of open data streams to improve arboviral surveillance nationwide, the current study represents a further step toward a data-driven, operational forecasting system. By demonstrating the practical application of these principles in the case of WNV, this work validates the strategic direction proposed by our group and supports its potential extension to other arboviruses such as Chikungunya. Despite these promising results, some limitations should be acknowledged. First, the reliance on open-access meteorological data and administrative-level aggregation may introduce some spatial imprecision, especially in areas with microclimatic variability. Second, while the model performs well retrospectively, prospective validation and real-time implementation will be key to fully assessing its public health utility and practical integration into early warning systems. Third, it would be important in future developments to include human mobility data within the model. Mobility patterns can influence the spatial dynamics of virus dispersion by facilitating the movement of infected hosts and vectors, which may result in the introduction of WNV into new regions or the amplification of transmission within endemic areas [1,29]. Incorporating these dynamics could significantly enhance the model’s spatial accuracy and forecasting power.

## Figures and Tables

**Table 1:** Clinical manifestations and risk factors of WNV infection in humans.

Category	Approximate Incidence Rate	Key Symptoms/Clinical Features	Associated Risks/Complications	Key Risk Factors
Asymptomatic	∼80% [5]	No symptoms	None directly	None specific
West Nile Fever (WNF)	∼20% [5]	Fever, headache, body aches (myalgias), rash, nausea/vomiting, diarrhea, arthralgia	Generally mild, self-limiting	General population exposure
West Nile Neuroinvasive Disease (WNND)	∼1% [5]			
Meningitis		Fever, headache, neck stiffness; CSF pleocytosis & elevated protein	Fatality rate up to 10% [6]	Age > 50 (10x risk), >80 (43x risk) [6]; Immunocompromised [5]; Chronic diseases (solid tumors, kidney disease) [7]
Encephalitis		Fever, altered mental status, seizures, focal neurological deficits, movement disorders (tremor)	Fatality rate up to 10% [5]; Long-term neurological impairment (fatigue, headaches, memory loss, muscle weakness, depression) [5]	Age > 50 (10x risk), >80 (43x risk) [5]; Immunocompromised [6]; Chronic diseases (solid tumors, kidney disease) [7]
Acute Flaccid Paralysis (AFP)		Isolated limb weakness/paralysis; poliomyelitis-like syndrome; damage to spinal anterior horn cells	Fatality rate up to 10% [5]; Potential for respiratory paralysis [6]; Little/no improvement on short-term follow-up [5]	Age > 50 (10x risk), >80 (43x risk) [5]; Immunocompromised [6]; Chronic diseases (solid tumors, kidney disease) [7]
Other Rare Complications	Rare [5]	Myocarditis, pancreatitis, fulminant hepatitis	Variable severity	None specific

**Table 2:** Performance metrics of the predictive model across the five most affected provinces in Italy.

Province	MAE	RMSE	R^2^	Median AE	Explained Variance	Pearson r	MAPE (%)	CRPS
Bologna	0.024	0.153	0.964	0.000	0.964	0.989	13.75	0.0123
Modena	0.036	0.334	0.896	0.000	0.897	0.979	13.83	0.0124
Venezia	0.005	0.051	0.986	0.001	0.986	0.996	5.21	0.0059
Padova	0.024	0.134	0.984	0.001	0.984	0.992	13.87	0.0139
Verona	0.004	0.023	0.996	0.001	0.996	1.000	6.35	0.0093

**Table 3:** Seasonal performance metrics (transmission vs. non-transmission) of the predictive model across the five most affected provinces in Italy.

Province	Season	MAE	RMSE	R^2^	MAPE (%)
Bologna	Transmission	0.034	0.182	0.9641	13.75
	Non-Transmission	0.000	0.000	0.0000	0.00
Modena	Transmission	0.051	0.396	0.8947	13.83
	Non-Transmission	0.000	0.000	0.0000	0.00
Venezia	Transmission	0.007	0.061	0.9855	5.21
	Non-Transmission	0.000	0.000	0.0000	0.00
Padova	Transmission	0.035	0.164	0.9834	13.87
	Non-Transmission	0.001	0.003	0.0000	0.00
Verona	Transmission	0.006	0.028	0.9963	6.35
	Non-Transmission	0.001	0.001	0.0000	0.00

**Table 4:** Top 10 features by SHAP importance in the XGBoost model.

Feature	SHAP Importance	Rank	Correlation with Cases
new_cases_diff_1	0.288694	1	0.713
new_cases_rolling_mean_7	0.042545	2	0.360
tmin	0.003564	3	0.109
new_cases_rolling_std_7	0.003114	4	0.358
rain	0.002972	5	−0.038
tmax	0.000096	6	0.106
new_cases_rolling_std_14	0.000060	7	0.254
new_cases_lag_7	0.000041	8	0.312
new_cases_diff_7	0.000037	9	0.285
sin_dayofyear	0.000033	10	0.012

## Data Availability

The data presented in this study are available at https://github.com/fbranda/west-nile, accessed on 10 September 2025. Epidemiological data were derived from the weekly bulletins published by the Italian National Institute of Health (ISS), which are publicly available at https://www.epicentro.iss.it/westnile/bollettino, accessed on 10 September 2025. Meteorological data were obtained from the Open-Meteo Archive API (https://open-meteo.com), accessed on 10 September 2025.

## Appendix A. Model Performance Metrics

### Appendix A.1. Mean Absolute Error (MAE)

The MAE measures the average magnitude of errors between predicted and actual values, offering an intuitive sense of typical prediction error in the same units as the target variable and is robust to outliers.

$$\mathrm{MAE}=\frac{1}{n}\sum_{i=1}^{n}\left|y_{i}-{\hat{y}}_{i}\right|$$

where $y_{i}$ is the observed number of WNV cases at time *i*, ${\hat{y}}_{i}$ is the corresponding predicted value, and *n* is the total number of observations.

### Appendix A.2. Root Mean Squared Error (RMSE)

The RMSE calculates the square root of the average squared differences, penalizing larger errors more heavily, thus highlighting significant deviations between predictions and observations.

$$\mathrm{RMSE}=\sqrt{\frac{1}{n}\sum_{i=1}^{n}{\left(y_{i}-{\hat{y}}_{i}\right)}^{2}}$$

### Appendix A.3. Coefficient of Determination ($R^{2}$)

The $R^{2}$ value indicates the proportion of variance in the observed data explained by the model, reflecting the overall goodness of fit and how well the model captures variability in the target variable.

$$R^{2}=1-\frac{\sum_{i=1}^{n}{\left(y_{i}-{\hat{y}}_{i}\right)}^{2}}{\sum_{i=1}^{n}{\left(y_{i}-\bar{y}\right)}^{2}}$$

where $\bar{y}$ is the mean of the observed values.

### Appendix A.4. Median Absolute Error (MedAE)

The MedAE reports the median of absolute prediction errors, providing insight into the typical error magnitude while minimizing the impact of extreme outliers.

$$\mathrm{MedAE}=\mathrm{median}\left(\left|y_{i}-{\hat{y}}_{i}\right|\right)$$

### Appendix A.5. Mean Absolute Percentage Error (MAPE)

The MAPE expresses the average prediction error as a percentage of actual values, facilitating interpretation relative to data scale and enabling comparison across different datasets, although it should be used cautiously when actual values $y_{i}$ approach zero.

$$\mathrm{MAPE}=\frac{100\%}{n}\sum_{i=1}^{n}\left|\frac{y_{i}-{\hat{y}}_{i}}{y_{i}}\right|$$

### Appendix A.6. Pearson Correlation Coefficient (r)

Assesses the strength and direction of the linear relationship between predicted and observed values, indicating how well model predictions follow the overall trend in the data without directly measuring error magnitude:

$$r=\frac{\sum_{i=1}^{n}\left(y_{i}-\bar{y}\right)\left({\hat{y}}_{i}-\bar{\hat{y}}\right)}{\sqrt{\sum_{i=1}^{n}{\left(y_{i}-\bar{y}\right)}^{2}}\cdot \sqrt{\sum_{i=1}^{n}{\left({\hat{y}}_{i}-\bar{\hat{y}}\right)}^{2}}}$$

where $\bar{y}$ and $\bar{\hat{y}}$ are the means of the observed and predicted values, respectively.

## Appendix B. Feature Description

**Table A1:** Overview of model features, their type, source, spatial and temporal resolution, and calculation or transformation method.

Feature	Type/Description	Source	Spatial Resolution	Temporal Resolution	Calculation/Transformation
new_cases	Raw target (daily cases)	ISS bulletins/aggregated file	Province/ administrative area	Weekly → daily	Sum of reported new cases per day aggregated at province level
target	Log-scaled target	Derived from new_cases	Province/ administrative area	Daily	Natural logarithm of daily new cases plus one to stabilize variance
tmax, tmin	Daily maximum/minimum temperature	Open-Meteo API	Grid aggregated to province	Daily	Average of all grid points within the province
rain	Daily precipitation (mean)	Open-Meteo API	Grid aggregated to province	Daily	Average daily precipitation over all grid points in the province
temp_avg	Daily mean temperature	Derived from tmax and tmin	Province	Daily	Mean of daily maximum and minimum temperature
temp_range	Diurnal temperature range	Derived from tmax and tmin	Province	Daily	Difference between daily maximum and minimum temperature
rain7d	7-day rolling mean precipitation	Derived from daily rain	Province	Daily (rolling)	Average precipitation over the previous 7 days
rain7d_sum	7-day cumulative precipitation	Derived from daily rain	Province	Daily	Sum of precipitation over the previous 7 days
sin_dayofyear, cos_dayofyear	Harmonic seasonality	Derived from date	N/A	Daily	Sine and cosine transformations to capture seasonal patterns
month, week, weekday, is_weekend	Categorical/time features	Derived from date	N/A	Daily	Extracted month, week number, weekday, and weekend indicator from date
new_cases_lag_7/14/21/28	Lagged cases	Derived from new_cases	Province	Daily	Number of new cases shifted by 7, 14, 21, and 28 days to capture temporal dependence
temp_avg_lag_7/14/21/28	Lagged mean temperature	Derived from temp_avg	Province	Daily	Daily mean temperature shifted by 7, 14, 21, and 28 days
new_cases_rolling_mean_7/14/21/28	Rolling mean of cases	Derived from new_cases	Province	Daily	Average number of new cases over rolling windows of 7, 14, 21, and 28 days
new_cases_rolling_std_7/14/21/28	Rolling standard deviation of cases	Derived from new_cases	Province	Daily	Standard deviation of new cases over rolling windows of 7, 14, 21, and 28 days
new_cases_diff_1, new_cases_diff_7	Short-term differences	Derived from new_cases	Province	Daily	Difference in new cases compared to previous day (1-day) and previous week (7-day) to capture short-term trends
